# The risks of ubiquinone and β-carotene deficiency and metabolic disorders in patients with oral cancer

**DOI:** 10.1186/s12885-020-06839-9

**Published:** 2020-04-15

**Authors:** Man-Yee Chan, Bor-Jen Lee, Po-Sheng Chang, Han-Yu Hsiao, Li-Ping Hsu, Chia-Hua Chang, Ping-Ting Lin

**Affiliations:** 1 Division of Oral and Maxillofacial Surgery, Department of Stomatology, Taichung Veterans General Hospital, Taichung, 407204 Taiwan; 2 School of Dentistry, College of Oral Medicine, Chung Shan Medical University, Taichung, 402367 Taiwan; 3 Department of Internal Medicine, Tungs’ Taichung Metro-Harbor Hospital, Taichung, 433402 Taiwan; 4 School of Medicine, Chung Shan Medical University, Taichung, 402367 Taiwan; 5 Department of Nutrition, Chung Shan Medical University, Taichung, 402367 Taiwan; 6 Graduate Program in Nutrition, Chung Shan Medical University, Taichung, 402367 Taiwan; 7 Department of Nutrition, Chung Shan Medical University Hospital, Taichung, 402367 Taiwan

**Keywords:** Ubiquinone, β-Carotene, Antioxidant vitamins, Metabolic disorders, Oral cancer

## Abstract

**Background:**

Cancer development is mediated by oxidative stress and inflammation, which may correlate with metabolic disorders. The aim of this study was to evaluate antioxidant vitamins status and metabolic parameters in patients with oral cancer according to tumor-node-metastasis (TNM) stages.

**Methods:**

A total of 194 patients with oral cancer were enrolled in this study. The patients were stratified for four groups according to cancer stages and that the statistics are comparisons across these groups. The levels of antioxidant vitamins (ubiquinone, β-carotene, vitamin A and E), metabolic parameters, oxidative stress, antioxidant enzymes activity, and inflammatory markers were measured.

**Results:**

More than half of the subjects had high blood pressure, central obesity, hyperglycemia, and hyperlipidemia regardless of TNM stage. With regard to antioxidant vitamins status, 46 and 94% of patients had β-carotene and ubiquinone deficiency, respectively. Patients in T3 and T4 stages had significantly lower antioxidant enzyme (catalase, *p* = 0.03) activity and higher inflammatory markers levels (high sensitivity C-reactive protein and interleukin-6, *p* < 0.01) than patients in the other stages. In addition, the level of β-carotene was negatively associated with waist circumference, and ubiquinone was positively associated with the level of high-density lipoprotein cholesterol (*p* < 0.05). Higher β-carotene and ubiquinone levels were negatively associated with hypertriglyceridemia and the risk of metabolic syndrome (*p* < 0.05).

**Conclusions:**

A high proportion of patients with oral cancer had ubiquinone or β-carotene deficiency and metabolic disorders. The level of ubiquinone or β-carotene was negatively associated with the risk of central obesity, hypertriglyceridemia, and metabolic syndrome. Since patients with oral cancer suffer from high oxidative stress and inflammation (particularly in the T3 and T4 stages), supplementation with antioxidant vitamins such as ubiquinone or β-carotene could be preferentially applied.

## Background

Current research has stated that tumor progression is related to changes in energy metabolism, resistance to apoptosis, and increased production of reactive oxygen species (ROS) [1, 2]. Higher levels of oxidative stress can then exacerbate the development of cancer [3], and ROS may also stimulate proinflammatory cytokine secretion to promote tumor metastasis and chronic inflammation [4]. Since an imbalance is found between ROS and antioxidant defense in oral cancer [5, 6], increasing oxidative stress and inflammation status may be harmful in regard to the prognosis of the disease.

Antioxidants play a critical role in cancer prevention by reducing oxidative stress and chronic inflammation [7]. It is well known that antioxidant vitamins such as vitamin A, E, or β-carotene can traverse the cell membrane because of their lipophilicity, neutralize ROS and reduce cellular oxidative damage [6, 8]. Recently, two case-control studies [9, 10] revealed that patients with oral cancer had significantly lower serum levels of the antioxidant vitamins: vitamin A, vitamin E, and β-carotene and noted that antioxidant vitamins could be predictors when monitoring the occurrence of oral cancer [9]. However, not all studies have obtained consistent findings [11, 12]. In addition to the antioxidant vitamins A and E, ubiquinone is also a powerful antioxidant in the mitochondria that participates in energy synthesis through the electron transport chain [13], and ubiquinone can regenerate vitamin E to fight free radicals with other antioxidants [14, 15]. However, few studies have investigated ubiquinone level in patients with oral cancer. Only one preliminary clinical study conducted by Folkers et al. in 1997 measured the ubiquinone levels in five head and neck cancer patients and found a subsequent ubiquinone deficiency, which may lead to biochemical dysfunction [16]. Recently, studies have indicated that antioxidant vitamins status may be related to the prevalence of metabolic syndrome [17–19]. According to a community-based health survey conducted in Taiwan, metabolic syndrome and unhealthy behaviors were associated with pre-oral cancerous lesions in adults [20]. Until now, few researchers have investigated the relationship between the status of antioxidant vitamins, such as ubiquinone, β-carotene, vitamin A and vitamin E, and metabolic disorders in patients with oral cancer. Thus, the aim of this study was to evaluate antioxidant vitamins status and metabolic parameters in patients with oral cancer according to tumor-node-metastasis (TNM) stages.

## Methods

### Subjects

This is a cross-sectional study. Patients who were over 20 years old and under 80 years old who were treated at a single medical center and who were diagnosed with oral cancer by stomatologist met the study entry criteria. The exclusion criteria included the following: 1) liver (hepatitis B or C), kidney or gastrointestinal diseases; 2) the use of antioxidant supplements; and 3) pregnant or lactating women. The study was approved by the Institutional Review Board of Taichung Veterans General Hospital, Taiwan. The subjects participated in the research after signing the informed consent form.

### Characteristics and anthropometrics

A questionnaire was used to understand the characteristics, including sex, age, lifestyle habits, and family medical history, of the subjects. The definitions for lifestyle habits as follows: smokers: individuals regularly smoking one or more cigarettes per day; for alcohol use: individuals regularly consuming one or more drink per day; betel nut use: individuals regularly consuming one or more betel nut per day. Exercise: individuals regularly exercising at least 3 times per week. The height, weight, and waist circumference of each subject were measured, and then the body mass index (BMI) was calculated. Central obesity was defined by a waist circumference ≥ 90 cm for male; ≥ 80 cm for female. Blood pressure was measured by an electronic sphygmomanometer, and a systolic blood pressure (SBP) ≥ 130 mmHg or a diastolic blood pressure (DBP) ≥ 85 mmHg was defined as high blood pressure.

### Biochemical measurements and pathological diagnosis

Subjects who were hospitalized and asked to fast for at least 8 h before surgical therapy, blood samples were collected in vacutainers containing K3-EDTA anticoagulant, sodium fluoride, or in the tube without anticoagulant. Then, these samples were centrifuged for 15 min to separate plasma, red blood cells (RBCs), and serum and stored at − 80 °C until analysis. After tumor resection surgery, the pathologist determined the pathological staging of the tumor in patients with oral cancer.

Fasting glucose was analyzed by an automated chemistry analyzer (Labospect, Tokyo, Japan), and a level ≥ 5.55 mmol/L was defined as hyperglycemia. Lipid profiles, including triglyceride (TG), low-density lipoprotein cholesterol (LDL-C), and high-density lipoprotein cholesterol (HDL-C), were analyzed by an automated chemistry analyzer (Hitachi 7070 & 7600, Tokyo, Japan), and hyperlipidemia was defined by TG ≥ 1.70 mmol/L, LDL-C ≥ 2.59 mmol/L, or HDL-C ≤ 1.04 mmol/L for male and < 1.30 mmol/L for female. High sensitivity C-reactive protein was measured by latex immunoassay. The level of IL-6 was analyzed by an enzyme-linked immunosorbent assay using commercial kits (R&D Systems Inc., Minneapolis, USA). The definition of high blood pressure, central obesity, hyperglycemia, and hyperlipidemia were according to the guidelines of the Administration of Health Promotion, Ministry of Health and Welfare, Taiwan (2007). The diagnostic criteria for metabolic syndrome in Taiwan are in accordance with the National Cholesterol Education Program Adult Treatment Panel III (ATP III, 2001), International Diabetes Federation (IDF, 2005), and the American Heart Association/National Heart, Lung, and Blood Institute (AHA/NHLBI, 2005) criteria [21–23].

### Oxidative stress and antioxidant enzymes measurements

The concentration of malondialdehyde was analyzed using the thiobarbituric acid colorimetric method [24]. The antioxidant enzyme activity of RBCs, including catalase, glutathione peroxidase, and superoxide dismutase, was determined by calculating the changes in the optical density of the enzyme activity reaction over one minute [25–27], and the data are shown as units/mg protein. RBC protein was analyzed using the bicinchoninic acid protein assay reagent kit (Thermo Scientific, Rockford, IL, USA).

### Antioxidant vitamins measurements

Antioxidant vitamins status was measured by high-performance liquid chromatography (HPLC). The measurement of ubiquinone was performed according to the method used by Littarru et al. [28]. The analysis column used was a LiChroCART®RP-18 (Merck, Germany), and the ultraviolet detector was set at 275 nm. The measurement of vitamins A and E was performed according to the method used by Karpińska et al. [29]. The analysis column for vitamin A and vitamin E analyses was a LiChrospher® 100 RP-18 (Merck, Germany), and the ultraviolet detector was set at 325 nm and 292 nm, respectively. The measurement of β-carotene was based on the method used by Kand’ár et al. [30]. The column for β-carotene analysis was a Purospher® STAR RP-18 (Merck, Germany), and the ultraviolet detector was set at 450 nm.

### Statistical analysis

All statistical tests in the study were conducted using SigmaPlot software (version 12.0, Systat, San Jose, California, USA). Descriptive statistics are presented as the mean ± standard deviation (median) or percentages. The normality of the distribution of the data was examined using the Shapiro-Wilk test. One-way ANOVA or Kruskal-Wallis test was used to examine the differences in antioxidant vitamins, metabolic parameters, oxidative stress, antioxidant enzymes activity, and inflammation according to the stage of the oral cancer; post hoc tests were further used to compare the differences among the stages. A chi-square test or Fisher’s exact test was used to compare the differences in categorical variables. Spearman’s rank order correlation coefficient was used to determine the correlations between antioxidant vitamins status and metabolic parameters in patients with oral cancer. The logistical regression analyses were used to examine the correlations between antioxidant vitamins and metabolic syndrome (the diagnostic criteria for metabolic syndrome were based on the guidelines of the Administration of Health Promotion, Ministry of Health and Welfare, Taiwan). The significance of level was set at a *p* value < 0.05.

## Results

The characteristics of the subjects according to TNM stages are shown in Table 1. Most patients with oral cancer were male (94%), and the mean age was 54 years. Notably, the subjects in the T4 stage had significantly lower waist circumference (*p* < 0.05), and slightly higher level of fasting glucose (*p* = 0.06) than patients in the other stages. Over 60 % of the subjects had current or ever smoking cigarette habits, alcohol use, or betel nut use, and subjects in the T4 stage had the highest proportion of smoking, alcohol consumption, and betel nuts use.

**Table 1:** Biochemical parameters and lifestyle characteristics of the subjects according to TNM stages

	T0 + 1 (***n*** = 45)	T2 (***n*** = 62)	T3 (***n*** = 30)	T4 (***n*** = 57)
Age (y)^1^	54.7 ± 10.1 (55.0)	54.5 ± 10.8 (55.5)	51.9 ± 8.4 (52.5)	54.6 ± 9.6 (54.0)
Males (n, %)	42 (93%)	57 (92%)	27 (90%)	57 (100%)
SBP (mmHg)	135.6 ± 21.2 (138.0)	137.8 ± 19.2 (138.5)	134.7 ± 17.8 (133.5)	132.2 ± 19.1 (130.0)
DBP (mmHg)	86.1 ± 13.1 (84.0)	88.3 ± 15.0 (86.0)	85.6 ± 10.9 (83.5)	86.7 ± 14.3 (87.0)
Waist (cm)	91.7 ± 9.6 (93.5)	94.8 ± 10.4 (93.8)	92.7 ± 9.9 (94.8)	88.9 ± 11.0 (89.0)*
BMI (kg/m^2^)	25.5 ± 4.0 (25.5)	27.0 ± 5.7 (26.3)	25.7 ± 4.2 (25.7)	25.8 ± 9.7 (24.5)
FG (mmol/L)	6.3 ± 2.1 (5.8)	7.4 ± 4.4 (5.8)	6.6 ± 2.8 (5.6)	7.6 ± 2.8 (6.5)
TC (mmol/L)	4.8 ± 0.9 (4.8)	4.9 ± 0.9 (4.6)	4.6 ± 1.0 (4.8)	4.5 ± 1.0 (4.5)
TG (mmol/L)	2.1 ± 1.4 (1.5)	2.6 ± 2.2 (1.9)	2.0 ± 2.0 (1.3)	2.3 ± 1.7 (1.7)
LDL-C (mmol/L)	2.9 ± 0.7 (2.8)	2.9 ± 0.9 (2.8)	2.7 ± 1.0 (2.9)	2.7 ± 0.8 (2.5)
HDL-C (mmol/L)	1.2 ± 0.3 (1.1)	1.1 ± 0.3 (1.0)	1.1 ± 0.5 (1.0)	1.1 ± 0.3 (1.0)
**Smoking cigarette**^**2**^				
Current (n, %)	19 (42%)	23 (37%)	12 (40%)	24 (42%)
Ever (n, %)	19 (42%)	28 (45%)	12 (40%)	29 (51%)
Never (n, %)	7 (16%)	11 (17%)	6 (20%)	4 (7%)
**Alcohol use**^**3**^				
Current (n, %)	12 (27%)	17 (27%)	8 (27%)	21 (37%)
Ever (n, %)	20 (44%)	23 (37%)	11 (37%)	21 (37%)
Never (n, %)	13 (29%)	22 (35%)	11 (37%)	15 (26%)
**Betel nut use**^**4**^				
Current (n, %)	8 (18%)	10 (16%)	7 (23%)	13 (23%)
Ever (n, %)	31 (69%)	48 (77%)	18 (60%)	38 (67%)
Never (n, %)	6 (13%)	4 (6%)	5 (17%)	6 (11%)
**Cigarette, alcohol, betel nut use**				
None (n, %)	1 (2%)	2 (3%)	3 (10%)	1 (2%)
One of them (n, %)	2 (4%)	6 (10%)	2 (7%)	4 (7%)
Both of them (n, %)	19 (42%)	19 (31%)	9 (30%)	14 (25%)
All above (n, %)	23 (51%)	35 (56%)	16 (53%)	38 (67%)
**Exercise**^**5**^**(n, %)**	20 (44%)	18 (29%)	13 (43%)	20 (35%)
***Family History***				
oral cancer (n, %)	1 (2%)	3 (5%)	1 (3%)	1 (2%)
Hypertension (n, %)	15 (33%)	18 (29%)	8 (27%)	15 (26%)
Diabetes (n, %)	7 (16%)	7 (11%)	8 (27%)	13 (23%)

^1^ mean ± SD (median), the differences in continuous variables were examined by One-way ANOVA or Kruskal-Wallis test; the differences in categorical variables were examined by chi-square test or Fisher’s exact test, * *p* < 0.05. ^2^smoker: individuals regularly smoking one or more cigarette per day. ^3^ alcohol use: individuals regularly consuming one or more drink per day. ^4^ betel nut use: individuals regularly consuming one or more betel nut per day. ^5^ exercise: individuals regularly exercising at least 3 times per week. TNM, tumor-node-metastasis

**Fig. 1:**
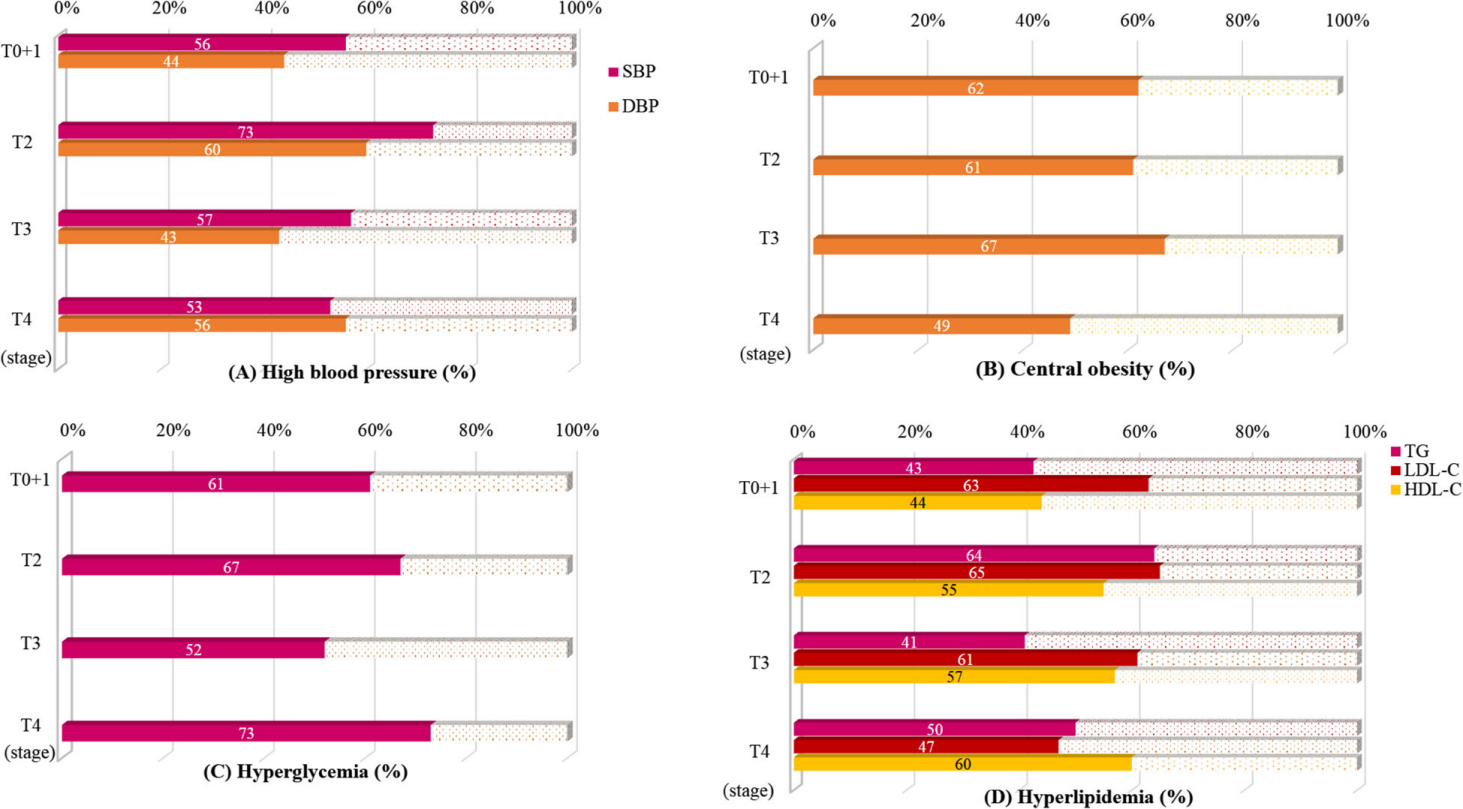
Prevalence of high blood pressure, central obesity, hyperglycemia, and hyperlipidemia in patients with oral cancer according to TNM stages. Descriptive statistics are presented as the percentages. **a** high blood pressure (SBP ≥ 130 mmHg; DBP ≥ 85 mmHg); **b** central obesity (waist ≥90 cm for male; ≥ 80 cm for female); (**c**) hyperglycemia (fasting glucose ≥5.55 mmol/L); **d** hyperlipidemia (TG ≥ 1.70 mmol/L; LDL-C ≥ 2.59 mmol/L; HDL-C ≤ 1.04 mmol/L for male and ≤ 1.30 mmol/L for female)

Figure 1 shows the prevalence of metabolic disorders in patients with oral cancer according to TNM stages. More than half of the subjects had high blood pressure, central obesity, hyperglycemia, and hyperlipidemia. Although not statistically significant, it is worth noting that a slightly lower proportion of patients in the T4 stage had central obesity (49%) and a higher proportion had hyperglycemia (73%) than patients in the other stages.

Table 2 shows the oxidative stress, antioxidant enzymes, and inflammation status of the patients with oral cancer according to TNM stages. The subjects in the T3 and T4 stages had significantly lower antioxidant enzyme (catalase, *p* = 0.03) activity than the subjects in the other stages. With regard to inflammation status, the subjects in the T4 stage had a significantly higher level of hs-CRP (*p* < 0.01), and the level of IL-6 was significantly higher in the subjects with T3 and T4 stages than in the subjects in the other stages (*p* < 0.01).

**Table 2:** Oxidative stress, antioxidant enzymes, and inflammation status of the subjects according to TNM stages

	T0 + 1 (***n*** = 45)	T2 (***n*** = 62)	T3 (***n*** = 30)	T4 (***n*** = 57)
***Oxidative stress***				
MDA (μM)	2.77 ± 0.95 (2.62)	3.10 ± 1.57 (2.81)	3.03 ± 1.09 (2.69)	2.63 ± 1.02 (2.41)
***Antioxidant enzymes***				
SOD (U/mg protein)	17.29 ± 7.11 (16.43)	16.68 ± 5.96 (16.31)	14.83 ± 5.91 (14.88)	16.53 ± 7.80 (15.65)
CAT (U/mg protein)	16.08 ± 6.71 (16.30)	16.03 ± 6.30 (14.56)	12.96 ± 5.39 (12.63)*	13.41 ± 4.75 (12.77)*
GPx (U/mg protein)	16.40 ± 6.34 (14.49)	16.23 ± 6.21 (15.18)	16.08 ± 5.64 (14.99)	14.81 ± 5.78 (13.72)
***Inflammation***				
hs-CRP (mg/L)	1.31 ± 2.81 (0.22)	0.47 ± 1.08 (0.16)	1.50 ± 2.96 (0.19)	2.49 ± 3.74 (0.82)*
IL-6 (pg/mL)	2.53 ± 3.30 (1.36)	1.96 ± 1.41 (1.45)	3.43 ± 3.20 (2.09)*	5.09 ± 4.39 (3.53)*

^1^ mean ± SD (median), the data were examined by One-way ANOVA or Kruskal-Wallis test, *p < 0.05. CAT, catalase activity; MDA, malondialdehyde; GPx, glutathione peroxidase; hs-CRP, high sensitivity C-reactive protein; IL-6, interleukin-6; SOD, superoxide dismutase; TNM, tumor-node-metastasis

**Fig. 2:**
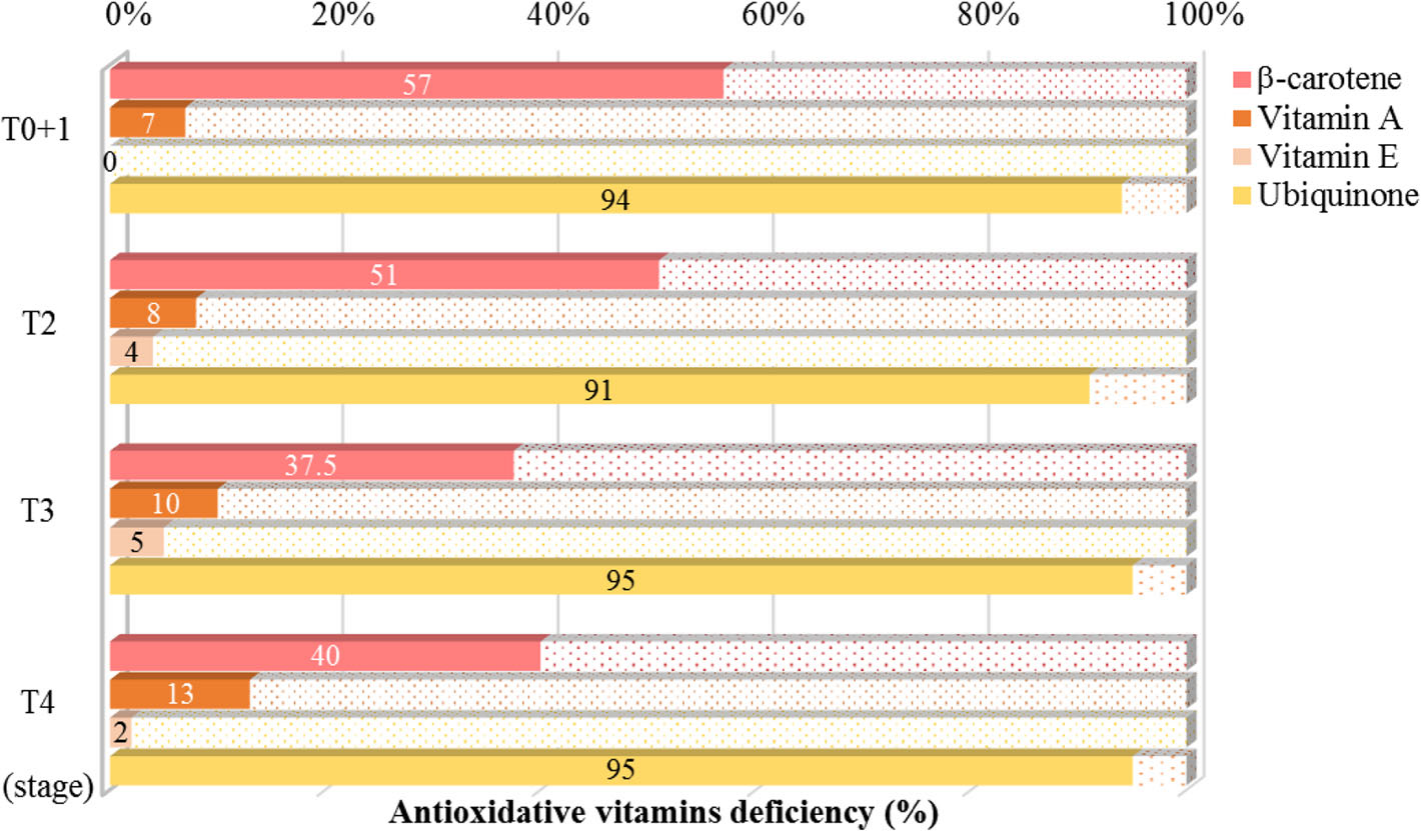
Prevalence of antioxidant vitamin deficiency^1^ in patients with oral cancer according to TNM stages. ^**1**^ Descriptive statistics are presented as the percentages. The definition of vitamins deficiency: plasma β-carotene < 200 nmol/L; vitamin A < 2.5 μmol/L; vitamin E < 11.6 μmol/L; ubiquinone < 500 nmol/L.

The prevalence of antioxidant vitamins deficiency in patients with oral cancer according to TNM stages is shown in Fig. 2. There was a high proportion (94%) of ubiquinone deficiency in patients with oral cancer regardless of TNM stage. More than half of the patients in the T0 + 1 and T2 stages had β-carotene deficiency; approximately 40% of patients in the T3 and T4 stages had β-carotene deficiency. Only 10% of patients among the four stages had vitamin A deficiency, and most patients with oral cancer did not have vitamin E deficiency.

The correlations between antioxidant vitamins and metabolic disorders or inflammation status in patients with oral cancer are shown in Table 3. The TNM stages were significantly positively correlated with metabolic disorders such as fasting glucose (*r* = 0.17, *p* < 0.05) and inflammation status (hs-CRP, *r* = 0.22; IL-6, *r* = 0.35, *p* < 0.05). Among the four antioxidant vitamins, β-carotene and ubiquinone status were significantly negatively correlated with the level of TG (β-carotene, *r* = − 0.20; ubiquinone, *r* = − 0.18, *p* < 0.05). In addition, the level of β-carotene and ubiquinone was significantly correlated with waist circumference (*r* = − 0.22, *p* < 0.05) and HDL-C level (*r* = 0.30, *p* < 0.05), respectively. Furthermore, we examined the correlations between β-carotene and ubiquinone status and the risk of metabolic syndrome (Table 4). Patients with higher β-carotene (β-carotene/LDL-C ≥ 120 nmol/mmol) or ubiquinone (ubiquinone/LDL-C ≥ 125 nmol/mmol) level had significantly lower risks of metabolic syndrome.

**Table 3:** Correlations between TNM stages, antioxidant vitamins, and blood pressure, waist circumference, glucose, lipid profiles, and inflammation status in subjects with oral cancer ^1^

	SBP (mmHg)	DBP (mmHg)	Waist (cm)	FG (mmol/L)	TG (mmol/L)	LDL-C (mmol/L)	HDL-C (mmol/L)	hs-CRP (mg/L)	IL-6 (pg/mL)
**TNM stages**	−0.09	0.01	−0.12	0.17*	− 0.01	− 0.10	− 0.11	0.22*	0.35*
**Antioxidant vitamins status**^**2**^									
β-carotene / LDL-C ≥ 81.3 nmol/mmol	0.02	0.01	−0.22*	0.02	−0.20*	−0.32*	− 0.05	0.08	0.06
Vitamin A / LDL-C ≥ 1.3 μmol/mmol	0.02	−0.04	− 0.08	0.06	0.13	−0.56*	0.01	−0.13	0.01
Vitamin E / LDL-C ≥ 9.5 μmol/mmol	0.13	0.13	−0.11	0.12	0.16	−0.45*	−0.09	0.07	0.17
Ubiquinone / LDL-C ≥ 102.0 nmol/mmol	0.08	0.03	0.00	0.07	−0.18*	−0.26*	0.30*	−0.01	− 0.03

^1^ The data in this table were analyzed by Spearman’s rank order correlation. *r*, Spearman correlation coefficients. ^2^ Antioxidant vitamins status were stratified by the median value of all subjects. *p < 0.05. DBP, diastolic blood pressure; FG, fasting glucose; HDL-C, high-density lipoprotein-cholesterol; hs-CRP, high sensitivity C-reactive protein; IL-6, interleukin-6; LDL-C, low density lipoprotein-cholesterol; SBP, systolic blood pressure. TG, triglyceride; TNM, tumor-node-metastasis

**Table 4:** Correlations between β-carotene and ubiquinone status and the risk of metabolic syndrome in subjects with oral cancer

	Metabolic syndrome ^**3**^	*p* values
	Odds ratios [95% confidence interval]	
**β-carotene / LDL-C**		
< 81.3 nmol/mmol	1.00	–
≥ 81.3 nmol/mmol ^**1**^	0.59 [0.29–1.19]	0.14
< 120 nmol/mmol	1.00	–
≥ 120 nmol/mmol ^**2**^	0.41 [0.20–0.85]	0.02
**Ubiquinone / LDL-C**		
< 102 nmol/mmol	1.00	–
≥ 102 nmol/mmol ^**1**^	0.67 [0.33–1.38]	0.28
< 125 nmol/mmol	1.00	–
≥ 125 nmol/mmol ^**2**^	0.42 [0.19–0.89]	0.02

^1^ The data in this table were analyzed by logistic regression. Antioxidant vitamins status were stratified by the median value of all subjects. ^2^ Antioxidative vitamins status were stratified by the median value of subjects without metabolic syndrome. ^3^ The diagnostic criteria for metabolic syndrome in Taiwan are based on the guidelines of the Administration of Health Promotion, Ministry of Health and Welfare, Taiwan. LDL-C, low density lipoprotein-cholesterol

## Discussion

In the present study, we found that more than half of the patients had high blood pressure, central obesity, hyperglycemia, and hyperlipidemia regardless of the TNM stage (Fig**.** 1). The prevalence of central obesity was lower in patients with T4 stage than in those in T0 to T3 stages. It seems that patients with T4 stage suffered from weight loss. Nutritional status may affect the prognosis and survival of patients, and a lower BMI (< 22.8 kg/m^2^) tended to increase the probability of death [31]. In fact, most of the subjects were overweight in the present study, and the BMI values ranged from 24.5–26.3. The mean waist circumference was higher than 90 cm in patients with stages T0 to T3. Although the values were lower in the stage T4, the mean waist circumference was still higher than 85 cm. As a result, patients with oral cancer in the present study had metabolic problems, particularly abdominal obesity and hypertriglyceridemia, which may affect the prognosis of the disease.

Recently, a case-control study conducted by Godala et al. revealed that patients with metabolic syndrome had a higher risk of antioxidant vitamins deficiency, particularly deficiencies in vitamin A, C, and E [18, 32]. In the present study, we found that a high proportion of patients with oral cancer had β-carotene and ubiquinone deficiency (Fig. 2) and these two antioxidants were significantly associated with waist circumference, lipid profiles (TG), and the risk of metabolic syndrome (Tables 3 and 4). Among these antioxidant vitamins, ubiquinone deficiency is most obvious in patients with oral cancer. The median level of ubiquinone was 280 nmol/L, which is lower than that of the healthy population (500–1700 nmol/L) [33]. Patients with cancer have been found to have a serious deficiency of ubiquinone [16, 34, 35]; in our previous clinical studies, we reported that patients with hepatocarcinoma had significantly lower ubiquinone concentration before or after surgery (before surgery: 320 nmol/L vs. after surgery: 280 nmol/L) [35]. Patients with oral cancer had lower ubiquinone concentration, which might be due to high oxidative stress [36]. Ubiquinone can act as a natural antioxidant, scavenging ROS during periods of elevated oxidative stress in patients with cancer [37]. In addition, it is interesting to note that the level of ubiquinone was positively correlated with the level of HDL-C (Table 3) and reduced the risk of metabolic syndrome (Table 4) in patients with oral cancer. Our previous clinical interventional studies demonstrated that ubiquinone supplementation could increase or maintain the level of HDL-C in patients with hepatocarcinoma or type 2 diabetes [37, 38]. Thus, administering antioxidants such as ubiquinone to oral cancer patients could be considered not only to reduce oxidative stress but also to regulate lipid metabolism.

Patients with malignancies had a lower β-carotene level, which has been found in previous studies [39, 40]. The consumption of β-carotene might relate to against free radicals [39] or low vegetables component diet [40]. According to a national survey from the US (NHANES 2001–2006), subjects with metabolic syndrome had significantly lower level of antioxidant vitamins (β-carotene and vitamin C) except vitamins A and E [17], which implies that antioxidant vitamins status may be related to dietary pattern. The limitation in the present study is that we did not investigate dietary intake; however, our previous study revealed that subjects with metabolic syndrome had a significantly higher intake of vitamin E and plasma vitamin E than those without metabolic syndrome, and the statistical significance disappeared after adjusting for lipid profiles [41]. We propose that this phenomenon, especially in the context of vitamin E, was due to the high consumption of fat, especially soybean oil, associated with metabolic syndrome [41]. Thus, patients with oral cancer rarely have vitamin A deficiency, and the fact that most of the subjects did not have vitamin E deficiency (Fig. 2) in the present study may be due to dietary factors. Vitamin A is found in large amounts in pig liver (114.96 mg retinal equivalents/g), sweet potato (15.20 mg retinal equivalents/g), sweet potato leaves (12.69 mg retinal equivalents/g), and carrots (99.80 retinal equivalents/g); these foods are very common in Taiwan. According to the latest report of the 2013–2016 National Nutrition Survey in Taiwan, Taiwanese adults rarely have vitamin A and vitamin E deficiency [42].

Inflammation and ROS are both key factors in carcinogenesis [7]. Patients with oral cancer have higher levels of oxidative stress regardless of stage. Our previous observational study investigated patients without metabolic syndrome and found that their median level of oxidative stress (MDA) was 1.94 μM. In this study, the median level of MDA was 2.61 μM in patients with oral cancer; obviously, patients with oral cancer suffer from high oxidative stress. In addition, a higher inflammation status was also observed in these patients, particularly those in the stage T3 and T4. Moreover, the antioxidant enzyme (CAT) activity of patients in the stage T3 and T4 was significantly lower than that of patients in stage T0 + 1 and T2 (Table 2). Inflammation has been shown to be associated with metabolic syndrome [43], and oral cancer patients with metabolic syndrome had higher inflammation status than those without metabolic syndrome (data not shown, hs-CRP, *p* = 0.02; IL-6, *p* = 0.01). Unhealthy behaviors, such as betel-nut chewing, smoking or alcohol consumption, may contribute to chronic inflammation, and increase the risk of metabolic syndrome [20, 44] In Taiwan, betel-nut chewing is the primary etiological factor in oral cancer development; 89% of the patients in this study had betel-nut chewing habit, followed by smoking (86%) and alcohol use (69%). These substances may stimulate the production of ROS or reactive nitrogen species, leading to an active inflammatory cascade [45] and increasing the risk of metabolic disorders. Based on the results of our study, we suggest that supplementation with antioxidant vitamins such as ubiquinone or β-carotene, could be used in preferentially for patients with oral cancer.

## Conclusions

This study is the first to investigate the antioxidant vitamins status in patients with oral cancer. In this cross-sectional study, we observed that a high proportion of patients with oral cancer had ubiquinone or β-carotene deficiency. Higher ubiquinone or β-carotene status was associated with reduced risk of central obesity, hypertriglyceridemia, and metabolic syndrome. Since patients with oral cancer suffer from high oxidative stress and inflammation, supplementation with antioxidant vitamins such as ubiquinone or β-carotene could be preferentially applied. Further interventional studies addressing metabolic issues are needed to clarify the significance of antioxidant vitamins in patients with oral cancer.

## Data Availability

The datasets generated and/or analyzed during the current study are available from the corresponding author on reasonable request.

## Abbreviations

AHA/NHLBI: American heart association/national heart, lung, and blood institute
ATP III: National cholesterol education program adult treatment panel III
BMI: Body mass index
CAT: Catalase activity
DBP: Diastolic blood pressure
FG: Fasting glucose
GPx: Glutathione peroxidase
HDL-C: High-density lipoprotein-cholesterol
hs-CRP: High sensitivity C-reactive protein
IDF: International diabetes federation
IL-6: Interleukin-6
LDL-C: Low density lipoprotein-cholesterol
MDA: Malondialdehyde
RBCs: Red blood cells
ROS: Reactive oxygen species
SBP: Systolic blood pressure
SOD: Superoxide dismutase
TG: Triglyceride
TNM: Tumor-node-metastasis
